# Mechanism of Enhanced *Bombyx mori* Nucleopolyhedrovirus-Resistance by Titanium Dioxide Nanoparticles in Silkworm

**DOI:** 10.1371/journal.pone.0118222

**Published:** 2015-02-18

**Authors:** Kaizun Xu, Fanchi Li, Lie Ma, Binbin Wang, Hua Zhang, Min Ni, Fashui Hong, Weide Shen, Bing Li

**Affiliations:** 1 School of Basic Medicine and Biological Sciences, Soochow University, Suzhou, Jiangsu 215123, P.R. China; 2 National Engineering Laboratory for Modern Silk, Soochow University, Suzhou, Jiangsu 215123, P.R. China; RMIT University, Australia

## Abstract

The infection of *Bombyx mori* nucleopolyhedrovirus (BmNPV) in silkworms is often lethal. It is difficult to prevent, and its lethality is correlated with both viral particle characteristics and silkworm strains. Low doses of titanium dioxide nanoparticles (TiO_2_ NPs) can promote silkworm growth and improve its resistance to organophosphate pesticides. In this study, TiO_2_ NPs’ effect on BmNPV resistance was investigated by analyzing the characteristics of BmNPV proliferation and transcriptional differences in silkworm midgut and the transcriptional changes of immunity related genes after feeding with TiO_2_ NPs. We found that low doses of TiO_2_ NPs improved the resistance of silkworm against BmNPV by 14.88-fold, with the mortalities of the experimental group and control group being 0.56% and 8.33% at 144 h, respectively. The proliferation of BmNPV in the midgut was significantly increased 72 h after infection in both experimental and control groups; the control group reached the peak at 120 h, while the experimental group took 24 more hours to reach the maximal value that was 12.63 times lower than the control, indicating that TiO_2_ NPs can inhibit BmNPV proliferation in the midgut. Consistently, the expression of the BmNPV-resistant gene *Bmlipase-1* had the same increase pattern as the proliferation changes. Immune signaling pathway analysis revealed that TiO_2_ NPs inhibited the proliferation of silkworm BmNPV to reduce the activation levels of janus kinase/signal transducer and activator of transcription (JAK/STAT) and phosphatidylinositol 3-kinase (PI3K)-Akt signaling pathway, while promoting the expression of *Bmakt* to improve the immunity. Overall, our results demonstrate that TiO_2_ NPs increase silkworm resistance against BmNPV by inhibiting virus proliferation and improving immunity in silkworms.

## Introduction

In many developing countries, such as China, India, Brazil, Vietnam and Thailand, sericulture is one of the main sources of income for farmers [1]. China’s raw silk production accounts for over 80% of the world total [2]. Among silkworm diseases that cause serious economic losses in sericulture, *Bombyx mori* nucleopolyhedrovirus (BmNPV) viral disease is the most serious one, thus constant research efforts have been devoted to this disease. However, no effective measures are currently available to stop the infection of BmNPV [3]. Improving silkworm’s resistance to BmNPV can help reduce the economic losses caused by this tough disease and promote the healthy development of sericulture [4].

BmNPV-resistance is mainly related to silkworm strains [5], and most strains are vulnerable to BmNPV infection. Among the few resistant strains, the silkworm strain KN has the highest resistance, while the strain 306 has the highest sensitivity [6, 7]. Traditional strain breeding has been tried to improve BmNPV-resistance in silkworms, but it takes several or even tens of years to finish, and the new strains obtained usually have low production performance. Therefore, it has become particularly important to search for an effective and simple method to enhance the resistance of all varieties of silkworm strains against BmNPV.

The spread of BmNPV in silkworm larvae is mainly by oral infection [1], and the main organ of invasion is the midgut, which is not only the place for digestion and absorption of nutrients but also the first barrier to defend against the invasion of foreign substances [8]. NPV infection in insects can activate the expression of certain genes [9], e.g. BmNPV can activate the endogenous antiviral protein Bmlipase-1 in silkworms, which as a result promotes strong resistance to BmNPV [10].

The janus kinase/signal transducer and activator of transcription (JAK/STAT) signaling pathway is an evolutionarily conserved innate immune pathway in the insect immune response mechanism [11, 12]. After the infection of *Autographa californica* nucleopolyhedrovirus (AcMNPV) in *Spodoptera frugiperda* Sf9 cells, the key gene *stat* in the JAK/STAT signaling pathway is activated to mediate the immune response against AcMNPV [13]. Xiao et al.’s study confirmed the activation of phosphatidylinositol 3-kinase (PI3K)-Akt pathway in sf9 cells after AcMNPV infection with increased phosphorylation of Akt [14]. Akt is the effector of PI3K, and the activation of PI3K leads to Akt activation, while the activation of Akt can be mediated through either PI3K-dependent or-independent mechanism [15, 16]. However, JAK/STAT and PI3K-Akt signaling pathways have not been reported in silkworms.

TiO_2_ NPs is the most widely used nanomaterial, especially in the purification of air, soil, and water [17–19]. TiO_2_ is a natural mineral oxide existing in three forms, anatase, rutile, and brookite. It is widely used in the industries of cosmetics, pharmaceuticals, food coloring, and implantable biomaterials, due to its suitable physical and chemical properties, such as its high stability making it a perfect choice for photocatalyst, antimicrobial agent, and preservative [20–23]. Becasue anatase TiO_2_ NPs were the most widely studied type, especially in silkworms, this study mainly focused on anatase TiO_2_ NPs.

It has been reported that low doses of TiO_2_ NPs (less than 200 μg/mL) do not have apparent toxicity to mammalian cells [24], bacteria [25], and animals [26]. Su et al. found that TiO_2_ NPs can protect the midgut of silkworm larvae against phoxim toxicity [27]. Li et al. have also shown that TiO_2_ NPs can ease the damages in silkworm silk gland and midgut caused by phoxim poisoning and improve cocooning rate [28, 29]. In addition, Zhang et al. found that TiO_2_ NPs can improve food conversion efficiency of five instar silkworm larvae and improve the quality of cocoon and silk [30]. Li et al. reported that feeding with TiO_2_ NPs can reduce the accumulation of reactive oxygen and NO after BmNPV infection, along with significantly enhanced expression of resistance related genes [31]. Investigations on the mechanism of TiO_2_ NPs’ effect on BmNPV proliferation focusing on JAK/STAT and PI3K-Akt signaling pathways have important significance.

## Materials and Methods

### Insects and Chemicals

The silkworm strain was Jingsong × Haoyue, and the BmNPV strain was T3 (GenBank:L3318), both of which were preserved in our laboratory.

The preparation of anatase TiO_2_ NPs was through controlling the hydrolysis oftitanium tetrabutoxide. The synthesis and characterization of TiO_2_ NPs were following the method described by Yang et al. [32, 33]. The average particle sizes of powders suspended in 0.5% w/v hydroxypropylmethylcellulose (HPMC) K_4_M solvent ranged from 5 to 6 nm after 12 h and 24 h incubation. As measured by DLS, themean hydrodynamic diameter of TiO_2_ NPs in HPMC solvent ranged from 208 to 330 nm (mostly 294 nm), and the zeta potentials after 12 h and 24 h incubation were 7.57 mV and 9.28 mV, respectively, and more detailed characterization of TiO_2_ NPs has been described by our team previously [33]. The morphology of the obtained TiO_2_ NPs was characterized by a transmission electron microscope (TEM) (Hitachi H-600, Japan). The detection of BmVPV in the hemolymph was carried by a scanning electron microscope (SEM).

TRIzol, chloroform, isopropanol, RNasin Inhibitor, dNTP, SYBR Premix, and other routine chemical reagents were all purchased from TAKARA Biotechnology (Dalian) Co., Ltd. Primers were synthesized by Shanghai Sangon Biological Technology and Services Co., Ltd.

### Silkworm Treatments

First to third instar larvae were reared with fresh mulberry leaves. From fourth instar, silkworms were reared in control or experimental zones, and each zone had 3 groups with 60 larvae in each group for the determination of morbidity and cocoon quality. The larvae of the experimental zones were continuously fed with TiO_2_ NPs at 5 mg/L [34] until mounting. The larvae of the control zones were fed with mulberry leaves which treated with sterile water. All treated leaves were dried before feeding for three times each day. From fifth instar, silkworms were fed with leaves with BmNPV (titer: 5.6 × 10^6^ polyhedral/mL). 100 g fresh mulberry leaves were dipped in BmNPV solution for 1 min and dried at room temperature before feeding. The rearing condition was long-day photoperiod (16: 8 h light/dark) at 25°C and approximately 70% relative humidity. After feeding with BmNPV, silkworms in both control zone and experimental zone were dissected to isolate midgut and fat body once every 24 h.

### Investigation of Biological Characteristics

Seven days after mounting, cocoon quality was surveyed by analyzing number of cocooning and non-cocooning, number of dead worm cocoons, whole cocoon mass, and cocoon shell mass. The cocooning rate, rate of death worm cocoons, and ratio of cocoon shell were calculated: cocooning rate (%) = number of cocooning / (number of cocooning + number of non-cocooning + number of dead worm cocoons) × 100; rate of death worm cocoons (%) = (number of dead worm cocoons / number of cocooning) × 100; ratio of cocoon shell (%) = (cocoon shell mass / whole cocoon mass) × 100.

### Detection of BmNPV Proliferation in Silkworm

For real-time detection of BmNPV proliferation in silkworms, mixed genomic DNA was extracted from midgut and BmNPV which in the midgut. Genomic DNA extraction was following the method described by Hughes et al. [35]. Quantitative real-time PCR (qPCR) primers were designed based on the sequences of polyhedrin genes *lef-1* and *gp64*. *Bmactin3* was used as the internal reference gene. To avoid the interference from RNAs, the primers were designed to target introns (Table 1). qPCR analysis was performed using the ViiA 7 Real-time PCR System [36] with SYBR Premix Ex *Taq* (Takara) following previously described method by our laboratory[37, 38].

**Table 1:** Sequences of primers used in quantitative RT-PCR.

Gene name	Forward primer(5′-3′)	Reverse primer(5′-3′)	Length (bp)
*Bmactin A3* (DNA)	GTTATCTGACGAATGACTTTGT	CGGAGTCCAGCACGATA	151
*Bmactin A3*(mRNA)	CGGCTACTCGTTCACTACC	CCGTCGGGAAGTTCGTAAG	147
*gp64*	CCGCTTCTTGACTCGGTGCT	ACCGTGGACACTGTGCTTCATC	243
*lef-1*	GGCGTCTACGACCCATTC	CAACGACAGCCGCAAGTA	172
*Bmlipase-1*	TGTTTTTGTCCACGGCT	TGGGAACTCCATTGACG	155
*Bmstat*	GAGCGTTATGGACGAGAAGC	CCTGGTTGCCGTGGACTATG	125
*Bmpi3k*	CTCATCAACATCAATGGCGACTA	CCCAGAGAAACTCCGAGCATAG	230
*Bmakt*	CGGGTTGCTGACCAAGGAC	CGGATTCCACTTGAGGCTTG	152

### Detection of Expression of Silkworm Anti-BmNPV Genes and Related Important Immune Signaling Genes

To explore the effects of TiO_2_ NPs on silkworm anti-BmNPV innate immune system, the endogenous BmNPV-resistance gene *Bmlipase-1* and the key genes of JAK/STAT and PI3K-Akt signaling pathways were selected for qPCR analysis. Total RNA was extracted from the midgut and fat body samples using Trizol reagent (Takara, Japan) and then treated with DNase to remove potential contamination from genomic DNA. RNA quality was assessed by formaldehyde agarose gel electrophoresis and was quantitated spectrophotometrically, and primer sequences showed in Table 1.

### Western Blot Analysis

Fat body samples of the control and TiO_2_ NPs treated groups were homogenized in lysis buffer supplemented with 1 mM of PMSF. The samples were centrifuged at 10,000 g for 10 min, and the supernatants were collected. The following procedure was carried out following Gu et al.[39]. A rabbit polyclonal phospho-Akt (Ser 505)-specific antibody or a rabbit polyclonal total Akt-specific antibody (Cell signaling, USA; 1:2000) was used as the primary antibody, and the HRP-conjugated goat anti-rabbit IgG (Santa Cruz Biotechnology, USA; 1: 5000) was used as the secondary antibody.

### Data Processing

Statistical analyses were conducted using the SPSS 19 software. Data are presented as mean ± standard error (SE). One-way analysis of variance (ANOVA) was carried out to compare the differences of means among multi-group data. Dunnett’s test was performed when each data set was compared with the solvent control data. Statistical significance for all tests was judged at a probability level of 0.05 (*P*<0.05).

## Results

### Characterization of TiO_2_ NPs

**Fig 1:**
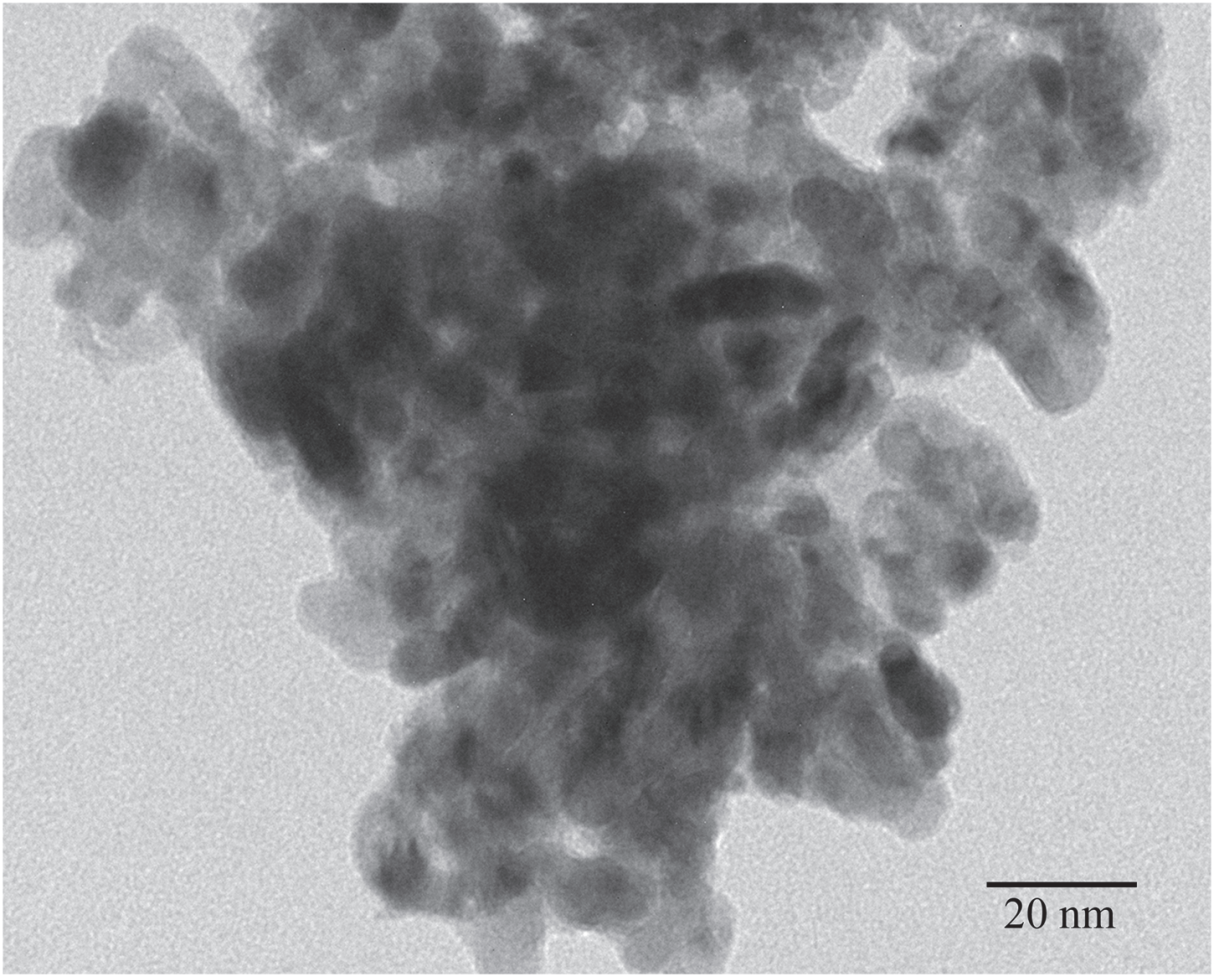
Transmission electron microscope (TEM) image of anatase TiO_2_ NPs particles.

The size of the TiO_2_ NPs was distributed from 5 to 6 nm as shown in the images of TEM (Fig. 1).

### TiO_2_ NPs Improves Silkworm Resistance to BmNPV

**Fig 2:**
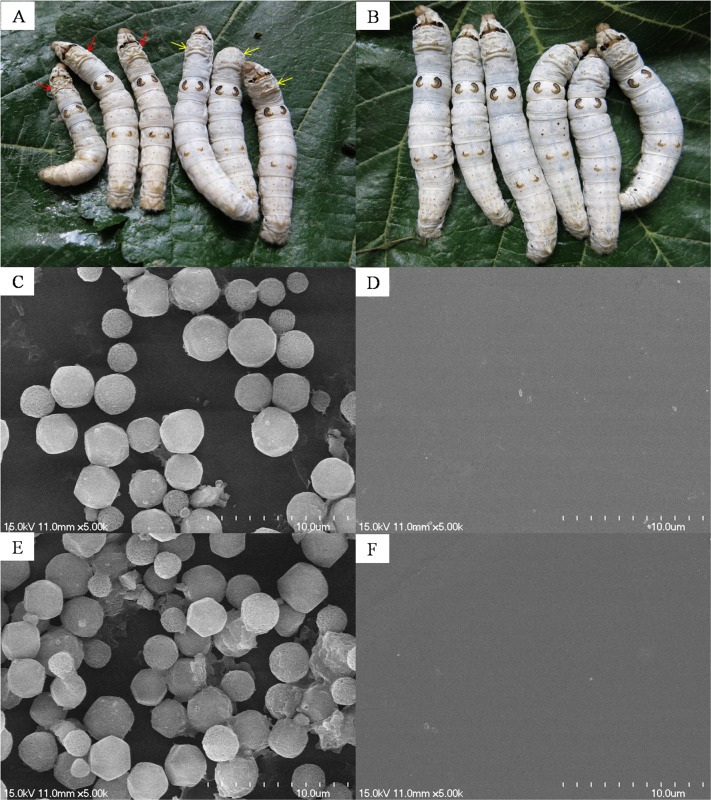
Detection of BmNPV in infected larvae and hemolymph. A, Silkworms in the control group at 120 h; red arrows point to the silkworms with apparent symptoms of BmNPV disease, and yellow arrows point to the silkworms without apparent symptoms of BmNPV disease. B, Silkworms in the experimental group at 120 h. C and E, and D and F represent SEM results of the control group and the experimental group at 120 and 144 h in the hemolymph of BmNPV, respectively.

After feeding with BmNPV for 120 h, some silkworms in the control group showed BmNPV disease symptoms, manifested as white body color, bulged intersegmental membrane, and manic crawling (Fig. 2A). As a contrast, silkworms in the experimental group grew well without disease symptoms (Fig. 2B). In order to detect the proliferation of BmNPV *in vivo*, 100 μL hemolymph was taken at 120 and 144 h for SEM analysis, respectively (Fig. 2C, E and D, F). BmNPV particles were observed in the control group, which were arranged on monolayer at 120 h but became aggregated at 144 h with apparently increased number of particles. In the experimental group, no BmNPV particles were observed at either 120 h or 144 h. These results indicated that TiO_2_ NPs significantly inhibited the proliferation of BmNPV in silkworm larvae.

**Fig 3:**
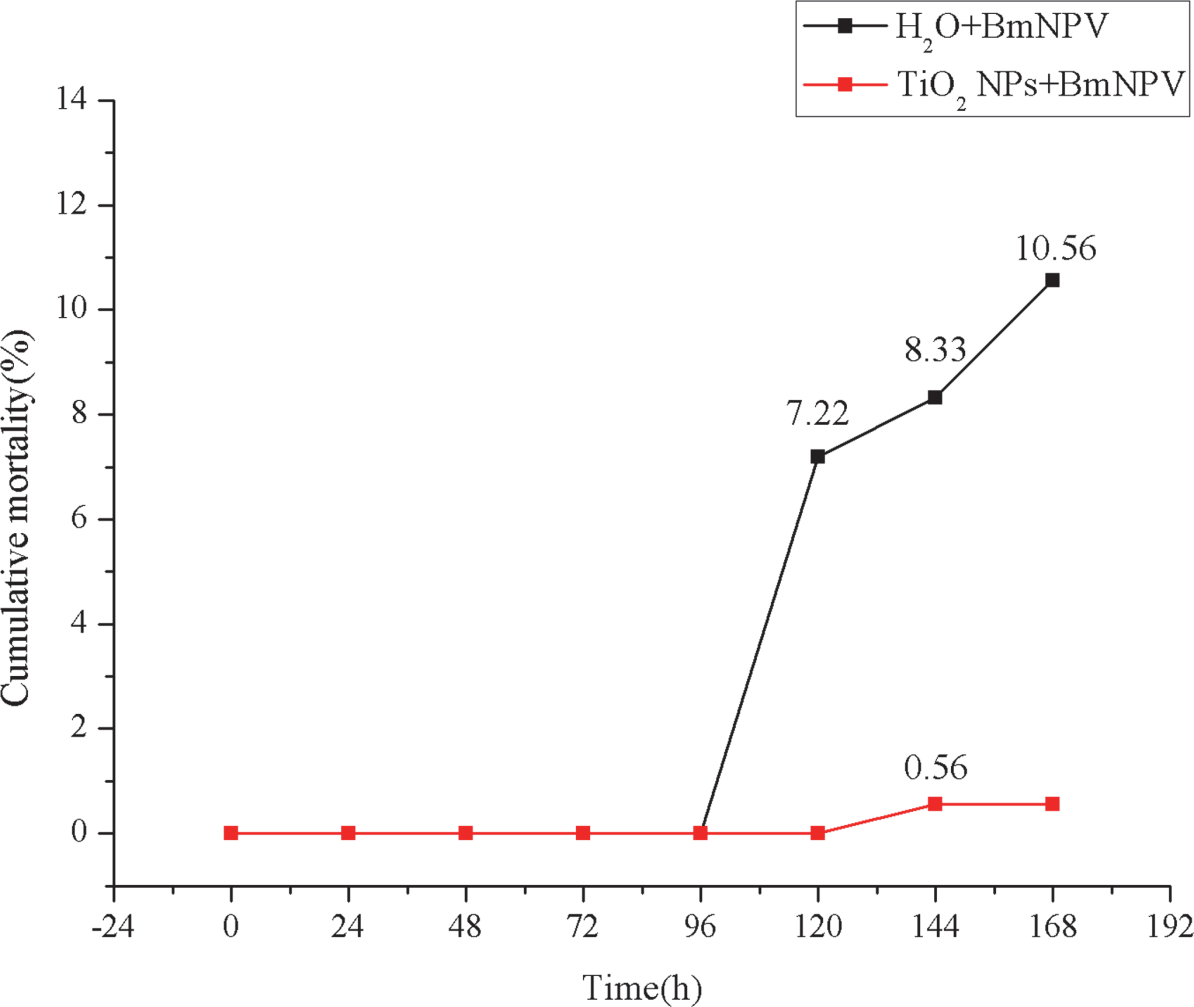
Cumulative incidence of BmNPV infection in silkworms. Black polylines represent the control group, and red polylines represent the experimental group. X-axis is the time after BmNPV infection, and Y-axis is the cumulative incidence (%).

As shown in Fig. 3, the average mortalities of larvae in the control group and the experimental group were 7.22% and 0%, respectively, at 120 h. Death of larvae was only observed at 144 h at a rate of 0.56%, while that of the control group reached 8.33%; at 168 h, the experimental group’s mortality was 0.56%, compared with the control group’s 10.56%. These results indicated that TiO_2_ NPs not only delayed the onset of BmNPV disease in silkworm larvae but also significantly reduced larvae mortality (14.88-fold increased in silkworm resistance against BmNPV).

As shown in Table 2, the larva survival rate of the experimental group at 99.44% ± 0.01% was significantly higher than that of the control group at 89.44% ± 0.02% (*P*<0.01); the cocooning rate of the experimental group was 49.13% ± 0.05%, significantly higher than the control group’s 40.94±0.04% (*P*<0.01). The rate of death worm cocoons of the experimental group was 38.51±0.05%, significantly lower than the control group’s 54.13±0.05% (*P*<0.05). In view of the survival rate and cocooning rate in H_2_O group was 100%, and there was no death worm cocoons, we mainly focused on the differences after NPV infection in the latter experiment. As shown in Table 3, the whole cocoon mass of the control group was 1.67±0.21 g, similar to the experimental group’s 1.73±0.19 g. The cocoon shell mass of the control group was 0.38 ± 0.009 g, significantly lower than that of the experimental group (0.41 ± 0.013 g) (*P*<0.05). The two groups’ ratio of cocoon shell were 22.94±2.80% and 23.82±2.66%, respectively, not significantly different from each other. These results indicated that feeding with TiO_2_ NPs significantly improved silkworm survival and cocoon and reduced the rate of death worm cocoons after BmNPV infection. Although TiO_2_ NPs did not significantly improve whole cocoon mass and ratio of cocoon shell, cocoon production was still significantly increased due to significantly improved silkworm cocooning rates that increased the total number of cocoons.

**Table 2:** Effect of TiO_2_ NPs on larval survival and cocooning after BmNPV infection.

	H_2_O	H_2_O+BmNPV	TiO_2_NPs+BmNPV	Ratio (%)
Survival rate of larvae (%)	100±0.00a	89.44±0.02a	99.44±0.01b	111.18
Cocooning rate (%)	100±0.00a	40.94±0.04a	49.13±0.05b	120.00
Death worm cocoons rate (%)	0±0.00a	54.13±0.05a	38.51±0.05b	71.14

The ratio represents the ratio of TiO_2_ NPs+BmNPV to H_2_O+BmNPV. Ranks marked with different letters mean they were significantly different at the 5% confidence level. Values represent means ± SD.

**Table 3:** Effect of TiO_2_ NPs on cocoon quality after BmNPV infection.

	H_2_O+BmNPV	TiO_2_NPs+BmNPV	Ratio (%)
Whole cocoon mass (g)	1.67±0.21a	1.73±0.19b	103.59
Cocoon shell mass (g)	0.38±0.009a	0.41±0.013b	107.89
Ratio of cocoon shell (%)	22.94±2.80a	23.82±2.66b	103.84

The ratio represents the ratio of TiO_2_ NPs+BmNPV to H_2_O+BmNPV. Ranks marked with different letters mean they are significantly different at the 5% confidence level. Values represent means ± SD.

### TiO_2_ NPs Affects BmNPV Proliferation in Silkworm Midgut

**Fig 4:**
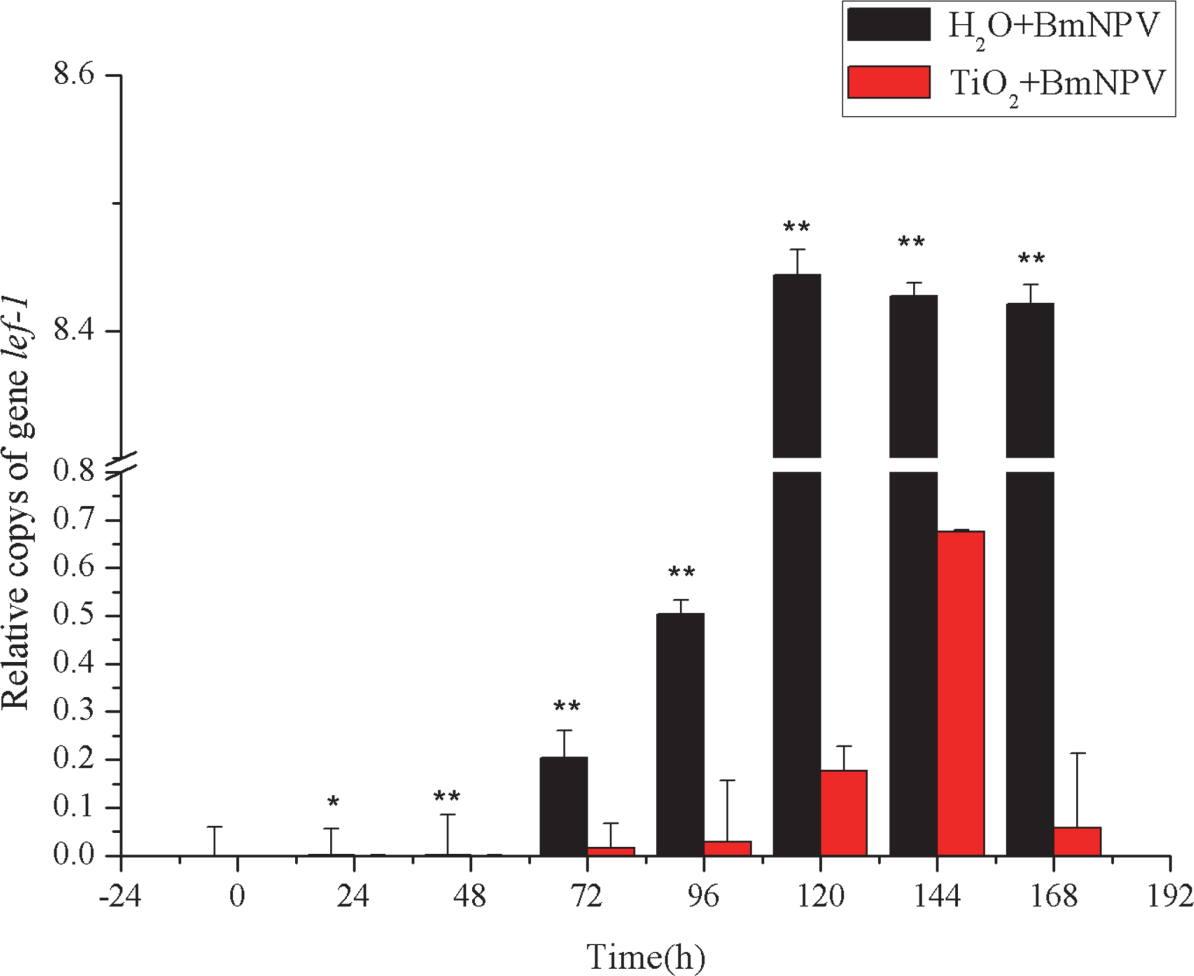
Relative copies of *lef-1*. Black and red histograms represent the control group and the experimental group, respectively. X and Y axes are the time after infection and the relative copies of BmNPV *lef-1*, respectively. The bars in the figure with different letters indicate statistically significant differences (*p*<0.05).

In order to detect BmNPV proliferation levels in silkworms accurately, genomic DNA was extracted from the mixture of silkworm midgut and BmNPV, and the relative copys of two essential genes for BmNPV amplification, *lef-1* and *gp64*, were selected as the detection indicators for qPCR analysis with *BmactinA3* as the internal reference gene. As shown in Fig. 4, the control group’s relative copys of *lef-1* was significantly higher than that of the experimental group. At 24 h and 48 h, *lef-1*’s relative copys were relatively low in both control and experimental groups, the control group’s were 12.69- and 13.02-fold of those of the experimental group, respectively. From 72 h, the relative copys of *lef-1* were apparently increased in both groups, and the control group entered a rapid increase period from 96 h to 120 h and reached the maximum at 120 h, and then maintained at a stable level; the experimental group’s rapid increase period was from 120 h to 144 h, and it reached the maximum at 144 h while reducing to 8.48% of the maximal level at 168 h. The peak value of the control group was 12.5-fold higher than that of the experimental group.

**Fig 5:**
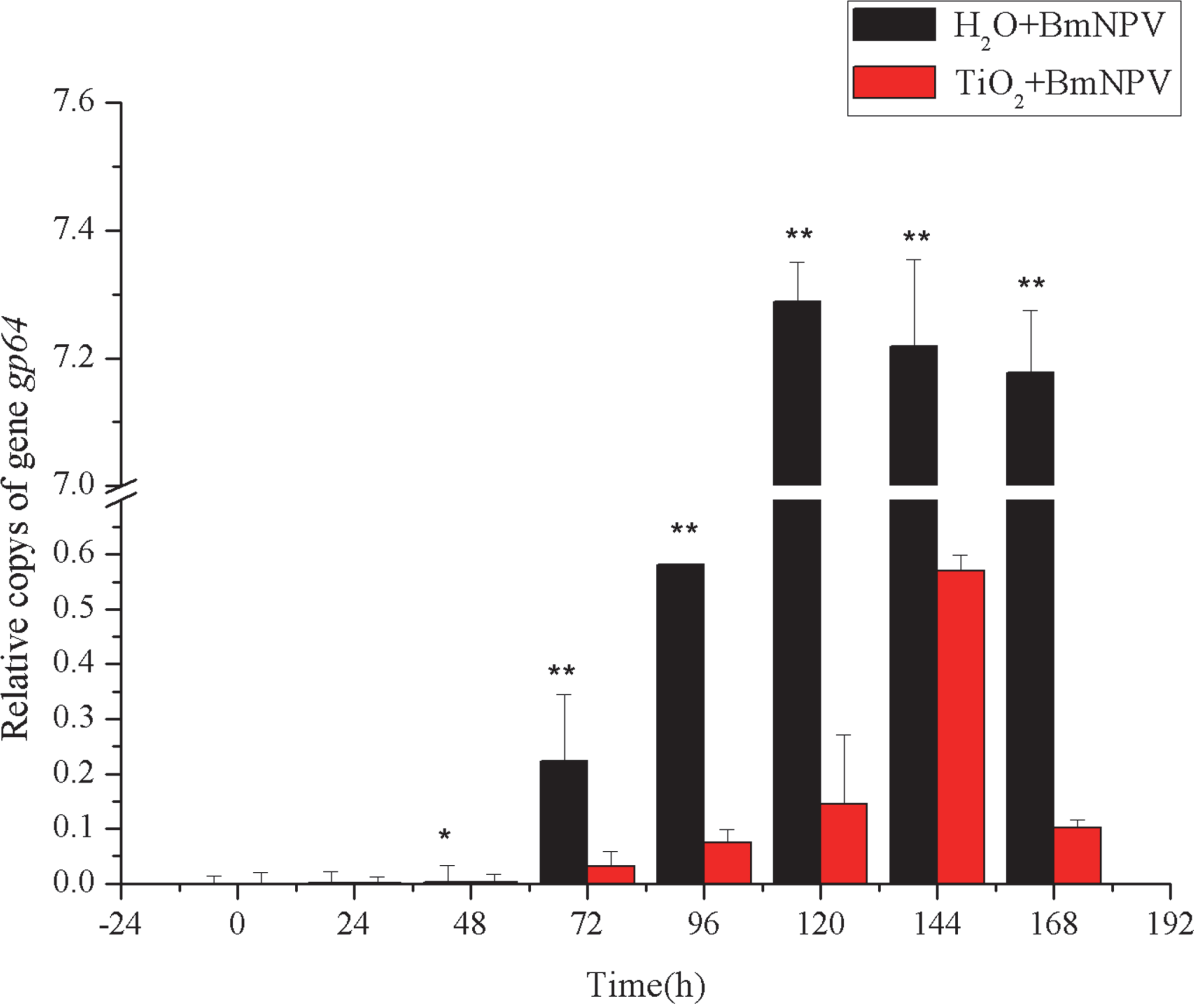
Relative copies of *gp64*. Black and red histograms represent the control group and the experimental group, respectively. X and Y axes represent the time after infection and the relative copy numbers BmNPV *gp64*, respectively. The bars in the figure with different letters indicate statistically significant differences (*p*<0.05).

BmNPV envelope protein gene *gp64* showed similar amplification pattern as that of *lef-1* (Fig. 5). The relative copys of *gp64* of the control group were all higher than those of the experimental group at all periods after BmNPV infection. 24 h and 48 h after the BmNPV infection, the relative copys of *gp64* were 2.18- and 1.13-fold of those of the experimental group, respectively. At 72 h, significant differences started to be observed, with the relative copys of *gp64* of control group showing 6.98-fold of the experimental group. Similar to the amplification of *lef-1*, *gp64* entered the rapid growth period also from 96 h to 120 h and reached the maximum at 120 h and maintained the level until mounting. In the experimental group, the rapid growth period was from 120 h to 144 h and the maximum value was reached at 144 h; it was reduced at 168 h to only 17.78% of the maximal level; the peak value of the control group was 12.76-fold higher than that of the experimental group.

Therefore, BmNPV proliferation in the control group experienced the classic latency period, rapid growth period, and plateau period; in each period, its amplification level was significantly higher than that of the experimental group; the control group entered the rapid growth period and reached the peak value both much more earlier than the experimental group. It indicated that TiO_2_ NPs inhibited the proliferation of BmNPV, delayed the emergence of the peak of virus proliferation, consistent with the results of larva morbidity. We also discovered that the amplifications of *lef-1* and *gp64* did not enter the plateau period after the peaks but were significantly decreased to 8.48% and 17.78% of the peak values, indicating that the inhibition of TiO_2_ NPs changed the proliferation trend of BmNPV in silkworm midgut.

### Transcriptional Characteristics of BmNPV-Resistance Relate Gene *Bmlipase-1*

**Fig 6:**
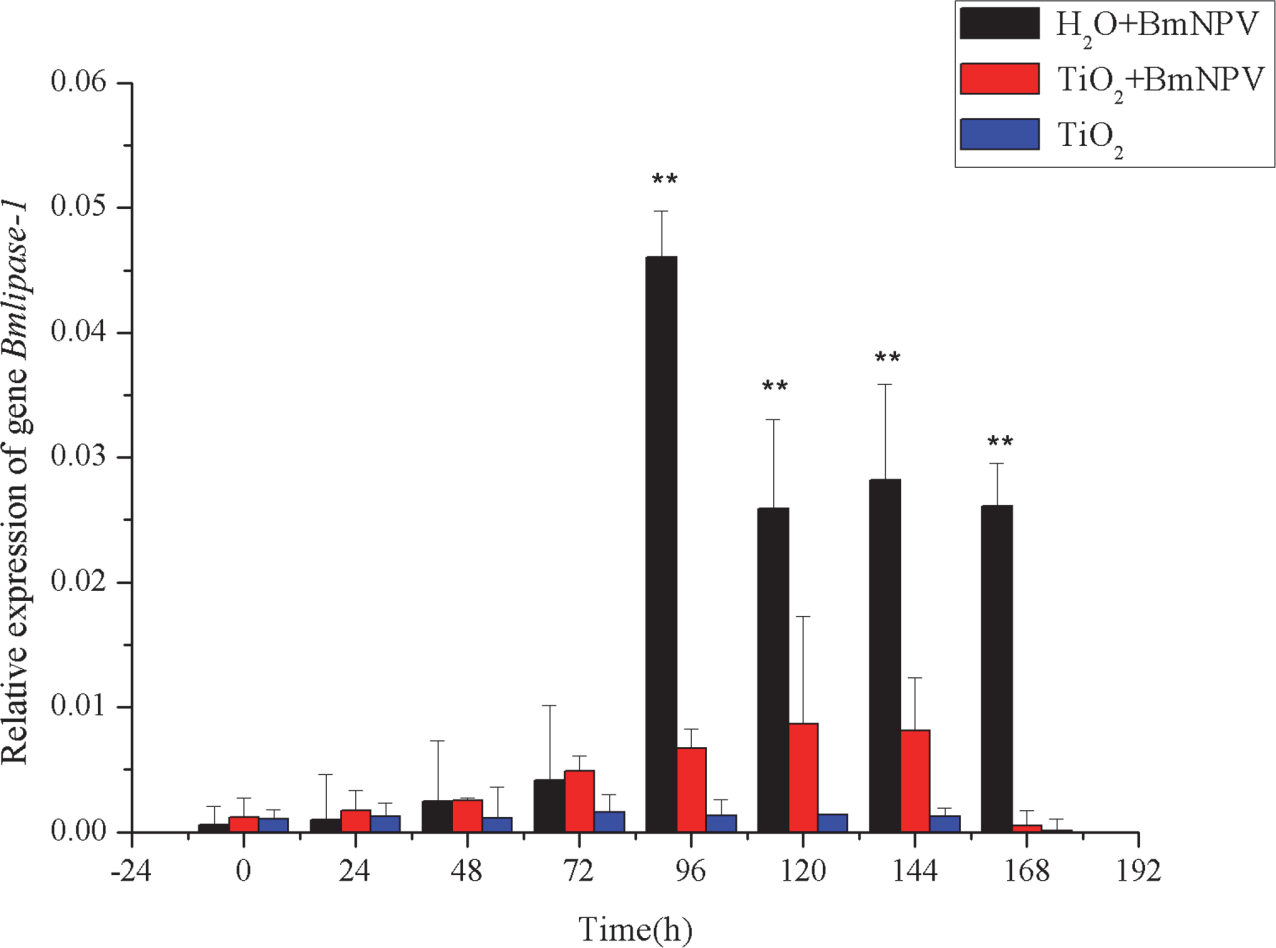
Relative expression of *Bmlipase-1*. Black and red histograms represent the control group and the experimental group, respectively, and blue histograms represent the TiO_2_ NPs-treated group. X and Y axes represent the time after infection and the relative expression of *Bmlipase-1*, respectively. The bars in the figure with different letters indicate statistically significant differences (*p*<0.05).

The transcription levels of *Bmlipase-1* in both experimental and control groups were measured in this study to investigate the effects of different titers of BmNPV on the induction of *Bmlipase-1* expression. As shown in Fig. 6, BmNPV infection led to mRNA levels of *Bmlipase-1* first increasing then decreasing in both groups. In the control group, its transcription level reached the peak at 96 h, while the experimental group had the maximum level at 120 h with only 18.7% of the control level. In addition, the control group’s peak value was maintained at about 3-fold of the experimental group’s since 96 h. The experimental group’s *Bmlipase-1* level reached the maximum at 120 h but decreased to the level similar to the initial infection period at 168 h. These results indicated that the transcription of *Bmlipase-1* was induced by BmNPV infection, and TiO_2_ NPs decreased the induction of *Bmlipase-1* by reducing the titer of BmNPV in silkworms. As a result, the occurrence of peak values was delayed by TiO_2_ NPs, which consistent with the changes in larva mortality. In addition, the peak values of *Bmlipase-1* transcription both occurred 24 h before the death of larvae. In the TiO_2_ NPs-treated group, the transcription level of *Bmlipase-1* showed no obvious changes, indicating that adding TiO_2_ NPs alone could not induce the expression of *Bmlipase-1*.

### Expression Characteristics of Key Genes in Immune Pathway

**Fig 7:**
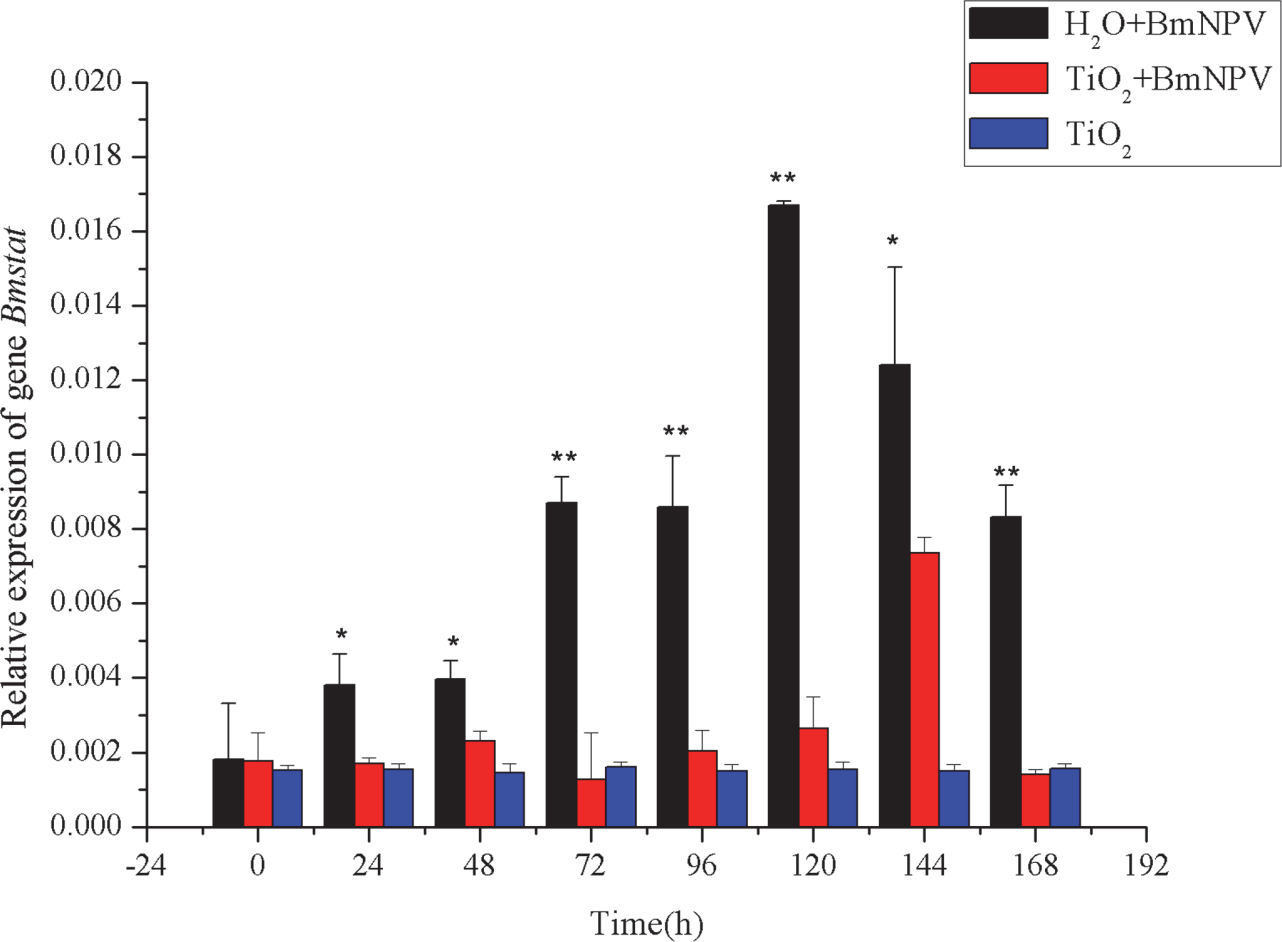
Relative expression of *Bmstat*. Black and red histograms represent the control group and the experimental group, respectively, and blue histograms represent the TiO_2_ NPs-treated group. X and Y axes represent the time after infection and the relative expression of *Bmstat*, respectively. The bars in the figure with different letters indicate statistically significant differences (*p*<0.05).

The resistance of silkworms against BmNPV is associated with not only resistance genes but also immune signaling pathways. In this study, the transcription levels of some key genes in the JAK/STAT and PI3K-Akt pathways were measured. The transcription levels of JAK/STAT pathway marker gene *Bmstat* were already upregulated at 24 h after BmNPV infection (Fig. 7), with the control group’s level being higher than the experimental group’s. The control group’s *Bmstat* transcription peaked at 120 h with 9.17 times of the level at 0 h. The transcription levels of *Bmstat* in the control group remained high level after reach the peak until mounting, which consistent with the trend of *Bmlipase-1* expression. In the experimental group, *Bmstat*’s relative transcription level remained low before 120 h, followed by increases until the peak at 144 h with 4.15-fold of the 0 h level. However, the peak value of *Bmstat*’s transcription level was only 44.04% of that of the control group. At 168 h, the relative transcription level of *Bmstat* was downregulated to 79.7% of the level at 0 h in the experimental group, while the relative level in the control group was 4.58-fold to the level at 0 h. Therefore, the infection of BmNPV activated the JAK/STAT immune signaling pathway in silkworms, and low titer of BmNPV delayed the activation of JAK/STAT immune signaling pathway and significantly reduced the expression of this pathway’s key gene, *Bmstat*. In the TiO_2_ NPs-treated group, the transcription level of *Bmstat* showed no obvious changes, indicating that adding TiO_2_ NPs alone could not induce the expression of *Bmstat*.

**Fig 8:**
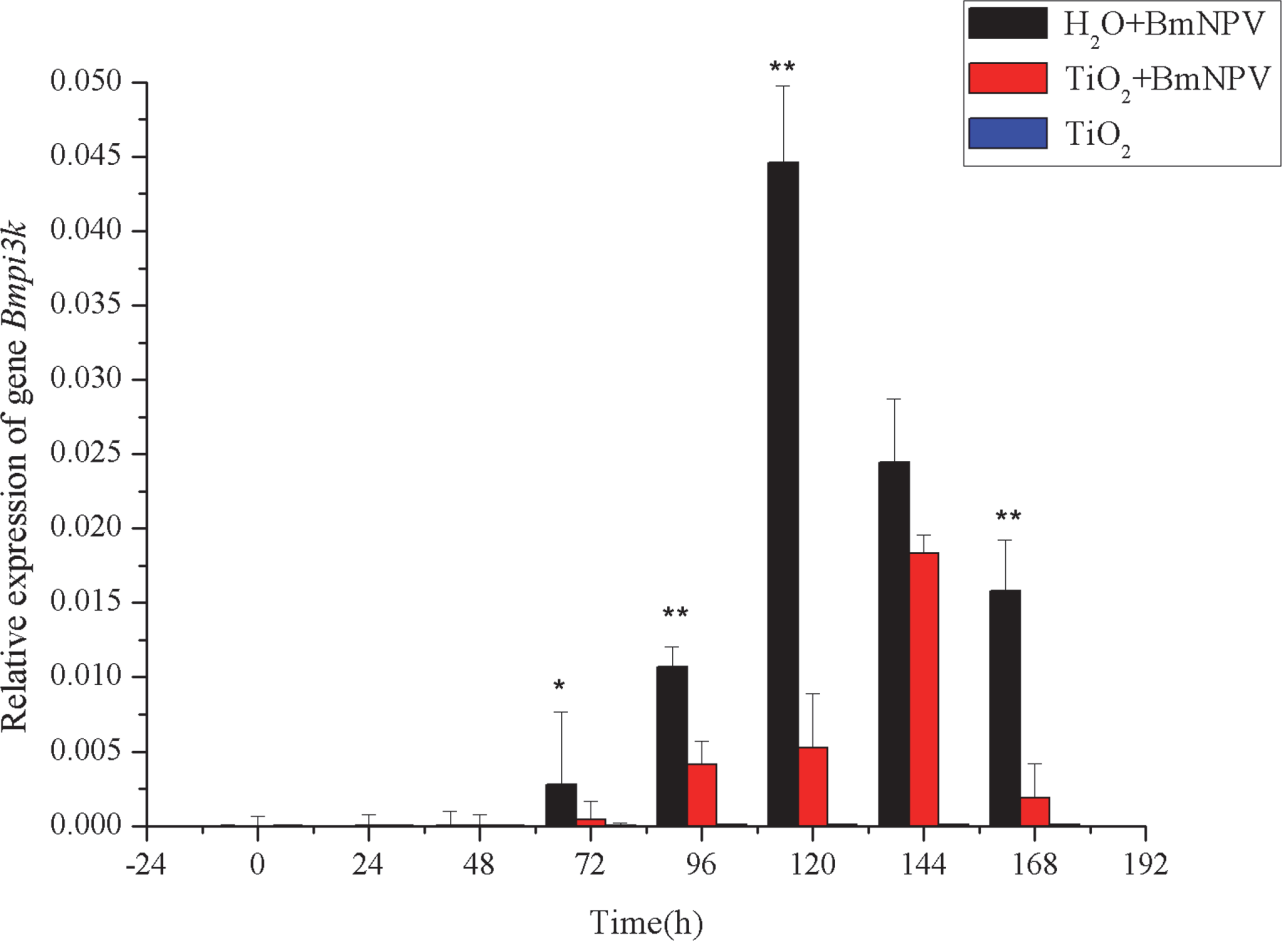
Relative expression of *Bmpi3k*. Black and red histograms represent the control group and the experimental group, respectively, and blue histograms represent the TiO_2_ NPs-treated group. X and Y axes represent the time after infection and the relative expression of *Bmpi3k*, respectively. The bars in the figure with different letters indicate statistically significant differences (*p*<0.05).

Besides JAK/STAT immune signaling pathway, PI3K-Akt pathway is also correlated with BmNPV infection in insects. It has been reported that PI3K-Akt pathway is required for the replication of baculoviruses, and *Bmpi3k* activation increases AcMNPV production [14]. In order to confirm the effects of TiO_2_ NPs on the PI3K-Akt signaling pathway response to BmNPV infection, the expression characteristics of *Bmpi3k* and *Bmakt* were examined in this study. As shown in Fig. 8, no *Bmpi3k* expression was detected 48 h after BmNPV infection; at 72 h, the control group showed upregulaton in *Bmpi3k* expression and achieved the maximum at 120 h, which was 15.99-fold of the level at 72 h; at 144 h and 168 h, its expression was downregualted to 8.77-fold and 5.66-fold of the level at 72 h, respectively. The relative expression levels of *Bmpi3k* of the experimental group started to show apparent increases at 96 h and reached the maximum at 144 h with 4.45-fold of the level at 96 h but only 41.09% of the control peak value; at 168 h, the level was reduced to 45.88% of 96 h’s level. These results indicated that the active response of PI3K-Akt signaling pathway by BmNPV infection in silkworms delayed the activation of *Bmpi3k* and reduced its activity by low titers of BmNPV. In the TiO_2_ NPs-treated group, no obvious changes were observed for the transcription level of *Bmpi3k*, indicating that adding TiO_2_ NPs alone could not activate the expression of *Bmpi3k*.

**Fig 9:**
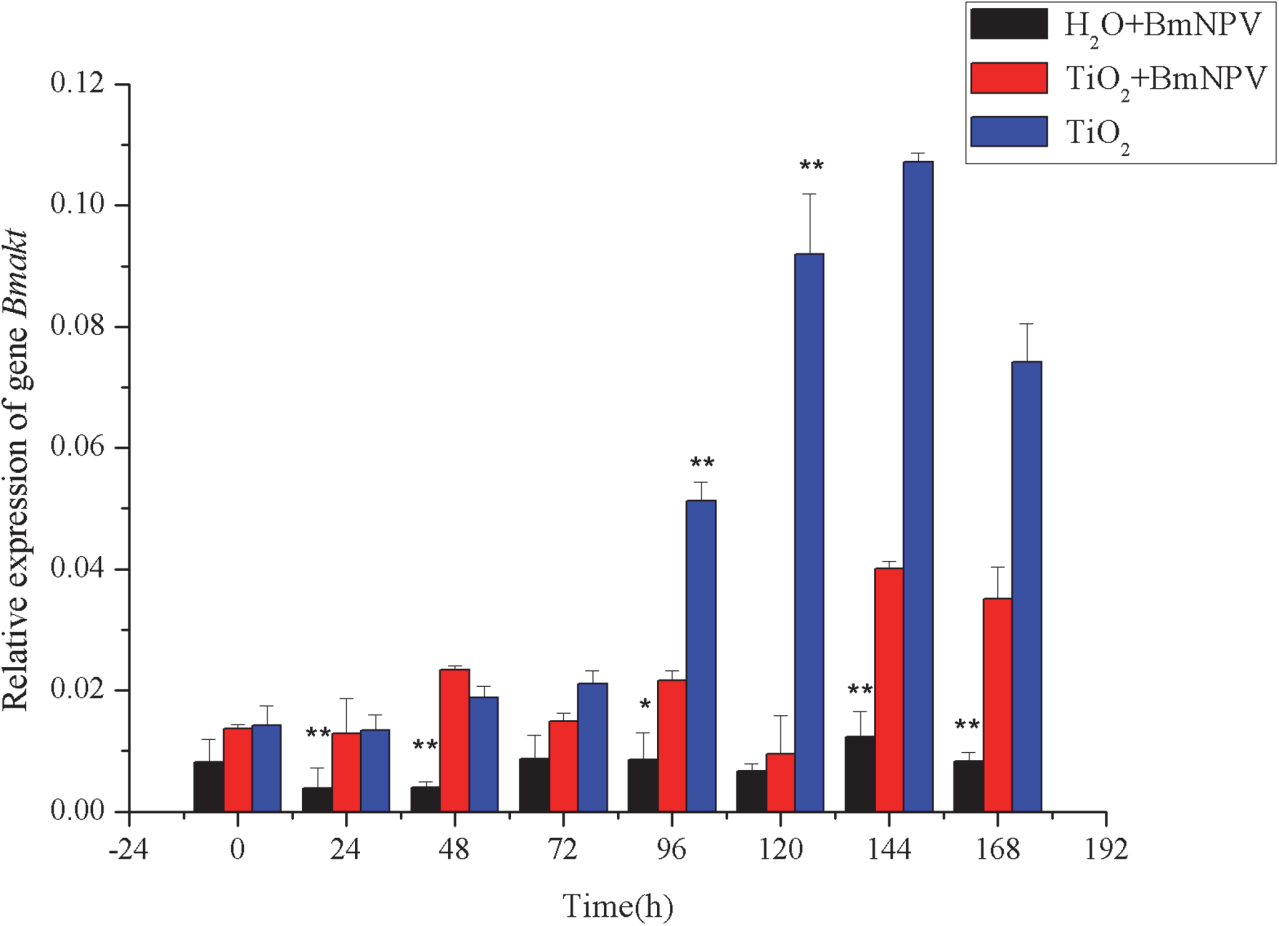
Relative expression of *Bmakt*. Black and red histograms represent the control group and the experimental group, respectively, and blue histograms represent the TiO_2_ NPs-treated group. X and Y axes represent the time after infection and the relative expression of *Bmakt*, respectively. The bars in the figure with different letters indicate statistically significant differences (*p*<0.05).

Akt is the effector of PI3K, and PI3K activation leads to Akt phosphorylation. However, Akt phosphorylation can be mediated through either PI3K-dependent or-independent mechanism. In order to verify whether *Bmakt* is activated, we examined the mRNA transcription level of *Bmakt* in silkworm midgut. As shown in Fig. 9, the experimental and TiO_2_ NPs groups’ *Bmakt* transcription levels were all higher than the control group’s at different time points. Without BmNPV infection at 0 h, the relative transcription level of *Bmakt* of the experimental group was higher than that of the control group and reached the maximum at 144 h after infection, which was 2.99-fold of the value at 0 h. In the TiO_2_ NPs-treated group, the transcription level of *Bmakt* showed significant increase and reached the maximum at 144 h; as shown in Fig. 8, *Bmpi3k* level started to be upregulated at 96 h and reached the highest value at 144 h, indicating that the upregulatoin of *Bmakt* was induced by TiO_2_ NPs, not by *Bmpi3k*. At 144 h, both *Bmakt* and *Bmpi3k’s* transcription levels reached the maximum, speculating that the upregulation of *Bmakt* was a joint effect of TiO_2_ NPs treatments and *Bmpi3k* activation.

### Effects of BmNPV and TiO_2_ NPs on Akt Phosphorylation

**Fig 10:**
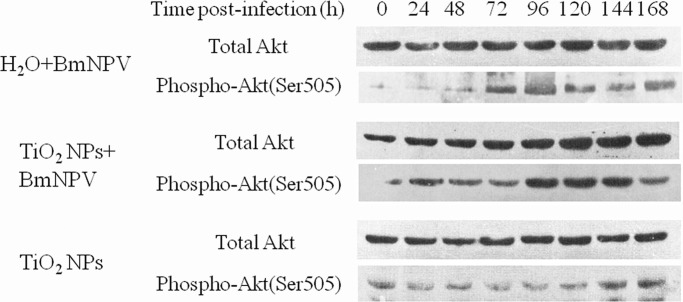
BmNPV infection and TiO_2_ NPs activate Akt phosphorylation. Fat body lysates isolated at the designated time points after infection were subjected to western blot analysis using a total Akt-specific or phospho-Akt (Ser 505) antibody.

Western blot was performed for fat body tissues. The total Akt was measured by an antibody recognizing total Akt, which demonstrated that the amount of total Akt protein remained stable throughout the infection (Fig. 10, upper panel). In contrast, an increased amount of total Akt protein was detected from 120 h after infection in TiO_2_ NPs treated group (Fig. 10, third panel). The phosphorylation of Akt was measured by an antibody that only recognizes Akt phosphorylation on Ser 505. As showed in Fig. 10 (second panel), the level of phosphorylated Akt in control group increased from 72 h after infection, clearing indicating that BmNPV infection induces Akt phosphorylation to resist the virus’s infection in silkworm fat body. In contrast, a high level of phosphorylated Akt was detected throughout the infection and without infection at 0 h in TiO_2_ NPs treated group (Fig. 10, fourth panel), especially at the time 96 to 144 h after infection, which consistent with the BmNPV proliferation characteristics of gene *lef-1* and *gp64* in silkworms. The bottom two panels represent the total Akt protein and phosphorylated Akt in TiO_2_ NPs-treated without BmNPV infection group, respectively, the result showed that both remained stable throughout the fifth instar. These results indicate that the upregulation of Akt phosphorylation was due to an upregulation of total Akt levels caused by the activation of upstream PI3K and TiO_2_ NPs treatments.

## Discussion

China’s annual production of cocoons is about 6.61×10^8^ kg; its sericulture farmers’ income is about 3.65 billion dollars and the annual total silk exports is about 33.22 billion dollars [1]. Each year, silkworm diseases may lead to about 20% economic losses [40], 80% of which was contributed by BmNPV disease. BmNPV has also become a serious threat to global sericulture [41]. Therefore, an effective method is urgently needed to improve BmNPV resistance. In recent years, nanoparticles have opened up new ways to treat viral diseases, and there had been reported that TiO_2_ NPs could ease silkworm injury caused by BmNPV [31, 42]. This study is the first one to reveal the inhibition of BmNPV proliferation in silkworms by TiO_2_ NPs and the activation of *Bmakt* in PI3K-Akt pathway that lead to improved resistance of silkworms to BmNPV. This provides a new method for BmNPV disease treatment.

Polyhedra occurred since 72 h after BmNPV infection, and traditional identification of polyhedra is through light microscopy with apparent lag and poor accuracy [43, 44]. In this study, qPCR was performed to detect BmNPV proliferation dynamics using genomic DNA extracted from silkworm midgut and midgut-BmNPV mixture for the first time. The differences in BmNPV copies could be detected as early as 24 h after infection, indicating the high sensitivity of qPCR that should improve early detection efficiency of BmNPV and provide a new idea for the research on the amplification of other viruses.

The proliferation characteristics of BmNPV in silkworm midgut indicated that TiO_2_ NPs can inhibit BmNPV proliferation in the organ (Fig. 4–5). Immune signaling pathway analysis revealed that BmNPV infection in silkworms led to upreguated transcription of both *Bmstat* and *Bmpi3k* (Fig. 6–8), indicating the activation of JAK/STAT and PI3K-Akt signaling pathways. Because the activation of *pi3k* may increase AcMNPV yield, and inhibits the transcription of it may reduce NPV production [14]. In the present study, TiO_2_ NPs effectively suppressed the upregulation of *Bmpi3k* transcription after BmNPV infection, which was probably the main reason for the reduction in BmNPV production. The relative transcription level of the downstream effector *Bmakt* was also detected, its transcription in the experimental group was not reduced along with the reduction of *Bmpi3k* levels and higher than that of the control group, indicating that *Bmakt* activation after BmNPV infection with TiO_2_ NPs was PI3K-independent. In addition, *Bmakt* expression in the experimental group was higher than that of the control group at 0 h (Fig. 9), confirming that TiO_2_ NPs can upregulate *Bmakt* expression. Studies have shown that Akt protein is a serine-threonine kinase that mediates the activities of other kinases, signaling proteins, and cell growth-, cell cycle-, and cell survival-associated transcription factors to improve immune response [45, 46], which is probably one of the reasons why TiO_2_ NPs improve silkworm production.

## Conclusions

In summary, the increased BmNPV-resistance in silkworms caused by TiO_2_ NPs was probably through the inhibition of BmNPV proliferation and the improvement in immune response. The inhibition of BmNPV proliferation was probably by decreasing the expression of *Bmpi3k*, and the enhanced immunity was through promoting *Bmakt* expression. However, the mechanisms behind these effects need further investigations.
